# High prevalence of plasmid-mediated 16S rRNA methylase gene *rmtB *among *Escherichia coli *clinical isolates from a Chinese teaching hospital

**DOI:** 10.1186/1471-2334-10-184

**Published:** 2010-06-23

**Authors:** Fang-you Yu, Dan Yao, Jing-ye Pan, Chong Chen, Zhi-qiang Qin, Chris Parsons, Le-he Yang, Qiao-qiao Li, Xue-qing Zhang, Di Qu, Liang-xing Wang

**Affiliations:** 1 Key Laboratory of Medical Molecular Virology of Ministries of Education and Health, Institute of Medical Microbiology and Institutes of Biomedical Sciences, Shanghai Medical School of Fudan University, Shanghai 200032, China; 2 Department of Respiratory Medicine, the First Affiliated Hospital of Wenzhou Medical College, Wenzhou325000, China; 3 Department of Intensive Care Unit, the First Affiliated Hospital of Wenzhou Medical College, Wenzhou325000, China; 4 Division of Infectious Diseases, Department of Medicine, Hollings Cancer Center, Medical University of South Carolina, Charleston, SC 29425, USA; 5 Department of Laboratory Medicine, the First Affiliated Hospital of Wenzhou Medical College, Wenzhou.325000, China

## Abstract

**Background:**

Recently, production of 16S rRNA methylases by Gram-negative bacilli has emerged as a novel mechanism for high-level resistance to aminoglycosides by these organisms in a variety of geographic locations. Therefore, the spread of high-level aminoglycoside resistance determinants has become a great concern.

**Methods:**

Between January 2006 and July 2008, 680 distinct *Escherichia coli *clinical isolates were collected from a teaching hospital in Wenzhou, China. PCR and DNA sequencing were used to identify 16S rRNA methylase and extended-spectrum β-lactamase (ESBL) genes, including *armA *and *rmtB*, and in situ hybridization was performed to determine the location of 16S rRNA methylase genes. Conjugation experiments were subsequently performed to determine whether aminoglycoside resistance was transferable from the *E. coli *isolates via 16S rRNA methylase-bearing plasmids. Homology of the isolates harboring 16S rRNA methylase genes was determined using pulse-field gel electrophoresis (PFGE).

**Results:**

Among the 680 *E. coli *isolates, 357 (52.5%), 346 (50.9%) and 44 (6.5%) isolates were resistant to gentamicin, tobramycin and amikacin, respectively. Thirty-seven of 44 amikacin-resistant isolates harbored 16S rRNA methylase genes, with 36 of 37 harboring the *rmtB *gene and only one harboring *armA*. The positive rates of 16S rRNA methylase genes among all isolates and amikacin-resistant isolates were 5.4% (37/680) and 84.1% (37/44), respectively. Thirty-one isolates harboring 16S rRNA methylase genes also produced ESBLs. In addition, high-level aminoglycoside resistance could be transferred by conjugation from four *rmtB*-positive donors. The plasmids of incompatibility groups IncF, IncK and IncN were detected in 34, 3 and 3 isolates, respectively. Upstream regions of the *armA *gene contained *IS*CR1 and *tnpU*, the latter a putative transposase gene,. Another putative transposase gene, *tnpD*, was located within a region downstream of *armA*. Moreover, a transposon, Tn*3*, was located upstream of the *rmtB*. Nineteen clonal patterns were obtained by PFGE, with type H representing the prevailing pattern.

**Conclusion:**

A high prevalence of plasmid-mediated *rmtB *gene was found among clinical *E. coli *isolates from a Chinese teaching hospital. Both horizontal gene transfer and clonal spread were responsible for the dissemination of the *rmtB *gene.

## Background

Aminoglycosides are clinically effective agents for treating a broad range of life-threatening infections caused by Gram-negative pathogens, usually in combination with β-lactam agents. However, increasing resistance to aminoglycosides is becoming a serious clinical problem [1-3]. Resistance to aminoglycosides is frequently due to the acquisition of modifying enzymes that vary in their substrate ranges, such as acetyltransferases, phosphorylases and adenylyltransferases [1,3]. Unlike modifying enzymes, 16S rRNA methylases have recently emerged as a novel mechanism for high-level resistance to all 4, 6-disubstituted deoxystreptamine aminoglycosides, such as arbekacin, amikacin, tobramycin, and gentamicin [4]. To date, six plasmid-encoded 16S rRNA methylases, including ArmA, RmtA, RmtB, RmtC , RmtD and NpmA, have been identified in clinical isolates of Gram-negative bacilli from multiple geographic locations [4-10]. *Escherichia coli *producing plasmid-mediated isolates extended-spectrum β lactamases (ESBLs) conferring to resistance to third-generation cephalosporins are mainly associated with hospital-acquired infections [11], but plasmid-mediated ArmA, RmtB and NpmA 16S rRNA methylases have been found in *E. coli *as well [5,10]. In China, although 16S rRNA methylases have been found among *E. coli *isolates from animals and humans [12-14], epidemiological data regarding 16S rRNA methylase production in *E. coli *clinical isolates are lacking. The aim of this study was to investigate the occurrence of 16S rRNA methylase genes in *E. coli *clinical isolates from a teaching hospital in Wenzhou, China.

## Methods

### Clinical isolates

Between January 2006 and July 2008, 680 non-duplicate *E. coli *clinical isolates were consecutively collected from a teaching hospital in Wenzhou, China. The clinical isolates were confirmed as *E. coli *using Gram stain and GNI cards on the VITEK-60 system (bioMe'rieux, Marcy l'E' toile, France). As plasmid-mediated 16S rRNA methylases can confer high-level resistance to most clinically important aminoglycosides, the isolates with concomitant resistance to gentamicin, amikacin, and tobramycin based on the criteria following the Clinical and Laboratory Standards Institute (CLSI) [15] were first screened for potential 16S rRNA methylase producers.

### Antimicrobial susceptibility testing

Initial antimicrobial susceptibilities were determined by using GNS cards on the VITEK-60 system (bioMe'rieux, Marcy l'E' toile, France). The antimicrobial susceptibilities of amikacin-, tobramycin-, or gentamicin-resistant isolates were further determined by the disk diffusion test using commercial disks for cefotaxime, ceftazidime, ciprofloxacin, levofloxacin, cefoxitin, imipenem, tetracycline and trimethoprim/sulfamethoxazole according to the criteria recommended by the CLSI [15]. MICs of amikacin, tobramycin, and gentamicin were further determined by the agar dilution method in accordance with the CLSI guidelines [15]. *E. coli *ATCC 25922 was used as quality control strain for antimicrobial susceptibility testing.

### Extraction of total DNA and plasmid DNA

Total DNA was extracted by boiling. Briefly, a fresh bacterial colony was suspended in 150 μL of sterile distilled water and boiled at 100°C for 10 min. After centrifugation at 15000 rpm for 15 min at 4°C, the supernatant was removed and stored at -20°C for further assays. Plasmid DNA of the donors, transconjugants and transformants was extracted with the QIAGEN (Hilden, Germany) Plasmid Midi kit according to the manufacturer's instructions. Plasmid size was estimated as previously described [16]. *E. coli *V517 and *E. coli *J53 containing plasmid R1 were used as a standard for plasmid size.

### Screening for 16S rRNA methylase genes

The *armA, rmtA, rmtB, rmtC*, *rmtD *, and *npmA *genes were detected by PCR with total DNA from potential 16S rRNA methylase producers and a series of primers as described previously [5,6,8-10]. The *Klebsiella pneumoniae *F25 harboring both *armA *and *rmtB *described in our previous study [17], was used as positive control in every test for detecting 16S rRNA methylase genes. All PCR products were directly sequenced on an ABI PRISM 3730 automated sequencer (Applied Biosystems, Foster City, CA). The nucleotide sequences were analyzed with software available over the Internet http://www.ncbi.nlm.nih.gov/.

### β lactamase characterization

All 16S rRNA methylase gene-positive isolates were tested for ESBL production by the CLSI-recommended confirmatory double disk combination test [15]. *K. pneumoniae *ATCC 700603 was used for the ESBL positive control. PCR was performed for the detection of β-lactamase genes in 16S rRNA methylase gene-positive isolates with the previously reported oligonucleotide primers, including *bla*_TEM_, *bla*_SHV _and *bla*_CTX-M _[18]. All PCR products were directly sequenced, and the sequences were compared with those in the GenBank nucleotide database at http://www.ncbi.nlm.nih.gov/blast/.

### Transfer of 16S rRNA methylase genes

To determine if aminoglycoside resistance was transferable in *E. coli *isolates with 16S rRNA methylase-bearing plasmids, a conjugation experiment was carried out in Luria-Bertani broth with *E. coli *J53 as the recipient as previously described [19]. Transconjugants were selected on tryptic soy agar plates containing sodium azide (100 μg/mL) for counterselection and amikacin (30 μg/mL) for plasmid-mediated aminoglycoside resistance selection. The plasmid extracts were electroporated into *E. coli *DH5α by using a Gene Pulser II apparatus (Bio-Rad, Hercules, CA), and the transformants were selected on Luria-Bertani agar plates containing amikacin (30 μg/mL).

### PCR-based replicon typing

All obtained transconjugants and transformants were subjected to typing by a PCR method described previously based on replicons of the major plasmid incompatibility groups among *Enterobacteriaceae *[20]. The plasmid DNA from the transconjugants and transformants was amplified by five multiplex and three simplex PCRs using 18 pairs of primers that recognized Inc replicons FIA, FIB, FIC, HI1, HI2, I1-I, L/M, N, P, W, T, A/C, K, B/O, X, Y, F, and FIIA.

### Southern hybridization

Southern hybridization was performed by standard methods with *armA*-and *rmtB*-specific digoxigenin (DIG)-labeled probes formed by the PCR DIG detection system (Roche Diagnostics GmbH). Briefly, plasmid DNA was subjected to electrophoresis in 0.7% agarose gels. After depurination, denaturation, and neutralization of the agarose gels, DNA was transferred to a positive-charged nylon membrane by capillary action. Plasmid DNA was hybridized successively with *armA*-specific and *rmtB*-specific DIG-labeled probes at 42°C under highly stringent conditions.

### Determination of flanking regions of the *armA *gene and the *rmtB *gene

To investigate the genetic environments of the *armA *and *rmtB *genes, the 54-kb plasmid of E31 transformant harboring the *armA *gene (pE31) and the other 54-kb plasmid of E35 transformant harboring the *rmtB *gene (pE35) were selected for DNA sequencing. Purified *armA*-and *rmtB-*bearing plasmids were directly sequenced by using a series of outwardly-directed primers specific to the locations near to the *armA *gene and the *rmtB *gene.

### PFGE

Chromosomal DNA was prepared from all 16S rRNA methylase gene-positive isolates and digested with 40 U *Xba*I (New England Biolabs, Beverly, MA). Electrophoresis was performed on 1.0% agarose gels in 0.5 M Tris/borate/EDTA buffer on a CHEF-Mapper XA PFGE system (Bio-Rad, Hercules, CA) for 22 h at 14°C, with run conditions of 6 V/cm, a pulse angle of 120° and pulse times from 5 to 20 s. A λDNA ladder (Amersham Biosciences, Piscataway, NJ) was used as molecular mass marker, and bands were stained with ethidium bromide (0.5 μg/mL) and photographed under UV light. Band profiles were interpreted by the criteria described previously [21]. The patterns with a difference of no more than three bands were considered to belong to the same type.

Ethical approval was not needed for the present study

## Results

### Prevalence of 16S rRNA methylase genes and antimicrobial susceptibility

Among tested 680 *E. coli *isolates, 357 (52.5%), 346 (50.9%) and 44 (6.5%)isolates were resistant to gentamicin, tobramycin and amikacin, respectively. All 44 amikacin-resistant isolates were concomitantly resistant to tobramycin and gentamicin. Thirty-seven of 44 (84.1%) amikacin-resistant isolates were positive for 16S rRNA methylase genes, among which 36 isolates were positive for *rmtB *gene and only one isolate was positive for *armA *gene. The *armA *and *rmtB *amplicons showed 100% identity with *armA *in *K. pneumoniae *BM4536 (GenBank AY220558) and *rmtB *in *Serratia marcescens *S-95 (GenBank AB103506). The *rmtA, rmtC*, *rmtD*, and *npmA *genes were not detected in any of the isolates tested. All 16S rRNA methylase gene-positive isolates were highly resistant to gentamicin, amikacin and tobramycin (MICs, ≥256 μg/mL) (Table 1). The positive rates of 16S rRNA methylase genes among overall isolates and amikacin-resistant isolates were 5.4% (37/680) and 84.1% (37/44), respectively. All 37 isolates harboring 16S rRNA methylase genes were resistant to ciprofloxacin, levofloxacin and trimethoprim/sulfamethoxazole but susceptible to imipenem. The sites of infection or colonization by 16S rRNA methylase gene-positive isolates from 37 patients were as follows: urine, 16 patients; pus, 10 patients; blood, 9 patients; sputum, 1 patient; and ascites, 1 patient. (Table 1).

**Table 1:** characteristics of the 37 *E. coli *isolates harboring 16S rRNA methylase genes and their transconjugants and transformants

Organism^a^	Origin	Ward^b^	PFGE	MIC (μg/ml)^c^			*rmtB*	*armA*	ESBL	Inc group	TEM	CTX-M
										
				GEN	AMK	TOB						
E1	Urine	11	A	>512	>512	>512	+	-	+		TEM-1	CTX-M-14

E1T				>512	512	512	+		+	F	TEM-1	CTX-M-14

E2	Pus	15	B	>512	512	512	+	-	+		TEM-1	CTX-M-14

E2T				512	256	512	+	-	+	F	TEM-1	CTX-M-14

E5	Urine	28	C	>512	512	256	+	-	+		TEM-1	CTX-M-15'

E5T				>512	256	256	+	-	+	N	TEM-1	CTX-M-15'

E6	Urine	11	D	>512	512	>512	+	-	+		TEM-1	CTX-M-15

E6T				256	256	512	+	-	+	F	TEM-1	CTX-M-15

E7	Pus	15	E	>512	>512	>512	+	-	-		TEM-1	-

E7T				512	256	256	+	-	-	F	TEM-1	-

E11	Blood	28	F	>512	512	>512	+	-	-		TEM-1	-

E11T				>512	512	512	+	-	-	F	TEM-1	-

E17	Urine	10	G	>512	>512	512	+	-	+		TEM-1	CTX-M-14/27

E17T				>512	512	512	+	-	+	N	TEM-1	CTX-M-14/27

E20	Blood	17	H	>512	>512	>512	+	-	+		TEM-1	CTX-M-15

E20C				>512	256	512	+	-	+	F	TEM-1	CTX-M-15

E21	Urine	11	H	>512	512	512	+	-	+		TEM-1	CTX-M-24

E21T				>512	256	256	+	-	+	F	TEM-1	CTX-M-24

E23	Urine	9	H	>512	512	>512	+	-	+		TEM-1	CTX-M-14

E23T				512	256	256	+	-	+	F	TEM-1	CTX-M-14

E24	Urine	11	H	512	256	512	+	-	+		TEM-1	CTX-M-14

E24T				512	256	256	+	-	+	F	TEM-1	CTX-M-14

E25	Blood	4	H	>512	>512	>512	+	-	+		TEM-1	CTX-M-14/15

E25T				512	512	512	+	-	+	F	TEM-1	CTX-M-14/15

E26	Pus	7	I	>512	512	512	+	-	-		TEM-1	CTX-M-14

E26T				512	256	256	+	-	-	F	TEM-1	CTX-M-14

E27	Pus	8	I	>512	>512	>512	+	-	+		TEM-1	CTX-M-14/15

E27T				>512	512	512	+	-	+	F	TEM-1	CTX-M-14/15

E28	Urine	11	J	512	512	512	+	-	+		TEM-1	CTX-M-14

E28T				256	256	256	+	-	+	F	TEM-1	CTX-M-14

E29	Urine	4	H	>512	512	>512	+	-	+		TEM-1	CTX-M-14/27

E29T				>512	256	512	+	-	+	F	TEM-1	CTX-M-14/27

E30	Blood	1	A	>512	512	>512	+	-	+		TEM-1	CTX-M-14/15

E30C				512	256	256	+	-	+	F, K	TEM-1	CTX-M-14/15

E31	Urine	15	K	>512	>512	>512	-	+	+		TEM-1	CTX-M-14

E31T				>512	256	512	-	+	+	F	TEM-1	CTX-M-14

E32	Blood	57	L	>512	256	512	+	-	-		TEM-1	CTX-M-15

E32T				>512	256	256	+	-	-	F	TEM-1	CTX-M-15

E33	Pus	21	H	>512	512	512	+	-	+		TEM-1	CTX-M-14

E33T				512	256	256	+	-	+	F, K	TEM-1	CTX-M-14

E34	Blood	26	A	>512	512	>512	+	-	+		TEM-1	CTX-M-14

E34T				>512	256	512	+	-	+	F	TEM-1	CTX-M-14

E35	Pus	11	M	>512	512	512	+	-	+		TEM-1	CTX-M-14

E35T				512	256	512	+	-	+	F	TEM-1	CTX-M-14

E36	Urine	11	N	>512	>512	>512	+	-	+		TEM-1	CTX-M-3

E36T				>512	512	512	+	-	+	F	TEM-1	CTX-M-3

E37	Urine	15	O	>512	512	512	+	-	-		TEM-1	-

E37T				512	256	256	+	-	-	F	TEM-1	-

E38	Urine	28	N	>512	>512	>512	+	-	+		TEM-1	CTX-M-14/15

E38T				>512	>512	>512	+	-	+	F	TEM-1	CTX-M-14/15

E39	Urine	56	P	512	256	512	+	-	+		TEM-1	CTX-M-14

E39T				256	256	256	+	-	+	F	TEM-1	CTX-M-14

E40	Pus	19	Q	>512	512	512	+	-	+		TEM-1	CTX-M-14

E40C				512	256	256	+	-	+	N	TEM-1	CTX-M-14

E41	Blood	2	R	512	256	512	+	-	-		TEM-1	-

E41T				512	256	256	+	-	-	F	TEM-1	-

E42	Pus	7	H	>512	>512	>512	+	-	+		TEM-1	CTX-M-14

E42T				>512	512	>512	+	-	+	F	TEM-1	CTX-M-14

E43	Pus	15	P	512	512	256	+	-	+		TEM-1	CTX-M-27

E43T				512	256	256	+	-	+	F	TEM-1	CTX-M-27

E44	Urine	9	H	>512	512	512	+	-	+		TEM-1	CTX-M-14/15

E44T				>512	256	256	+	-	+	F	TEM-1	CTX-M-14/15

E45	Urine	11	G	512	256	256	+	-	+		TEM-1	CTX-M-14/15

E45T				256	256	256	+	-	+	F	TEM-1	CTX-M-14/15

E46	Sputum	54	H	>512	256	512	+	-	-		TEM-1	-

E46T				512	256	256	+	-	-	F	TEM-1	-

E47	Blood	25	S	>512	512	512	+	-	+		TEM-1	CTX-M-14/15

E47T				>512	256	512	+	-	+	F	TEM-1	CTX-M-14/15

E48	Blood	9	N	>512	512	>512	+	-	+		TEM-1	CTX-M-14

E48C				>512	256	512	+	-	+	F, K	TEM-1	CTX-M-14

E49	Pus	7	H	>512	512	512	+	-	+		TEM-1	CTX-M-14

E49T				>512	256	256	+	-	+	F	TEM-1	CTX-M-14

E51	Ascites	6	H	>512	512	512	+	-	-		TEM-1	-

E51T				512	256	256	+	-	-	F	TEM-1	-

a; T, transformant; C, transconjugant b;11, 15, 28, 10, 17, 9, 4, 7, 8, 1, 57, 21, 26, 56, 19, 2, , 54, 25 and 6 represent different clinical units. C; GEN, gentamicin; AMK, amikacin; TOB, tobramycin

### β lactamase characterization

Among 37 isolates harboring 16S rRNA methylase genes, 29 isolates (78.4%, 29/37) were found to coproduce ESBLs. Thirty-one isolates were found to harbor *bla*_CTX-M_, among which 24, 11, 3, 1 and 1 isolates were positive for *bla*_CTX-M-14_, *bla*_CTX-M-15_, *bla*_CTX-M-27_, *bla*_CTX-M-3 _and *bla*_CTX-M-24_, respectively. Seven of 31 *bla*_CTX-M_-positive isolates harbored both *bla*_CTX-M-14 _and *bla*_CTX-M-15_, and two isolates harbored both *bla*_CTX-M-27 _and *bla*_CTX-M-14 _(Table 1). All 16S rRNA methylase gene-positive isolates were positive for *bla*_TEM_, and all *bla*_TEM _type genes were identified as the narrow spectrum β-lactamase gene, *bla*_TEM-1._

### Transfer of aminoglycoside resistance and plasmid analysis

**Figure 1:**
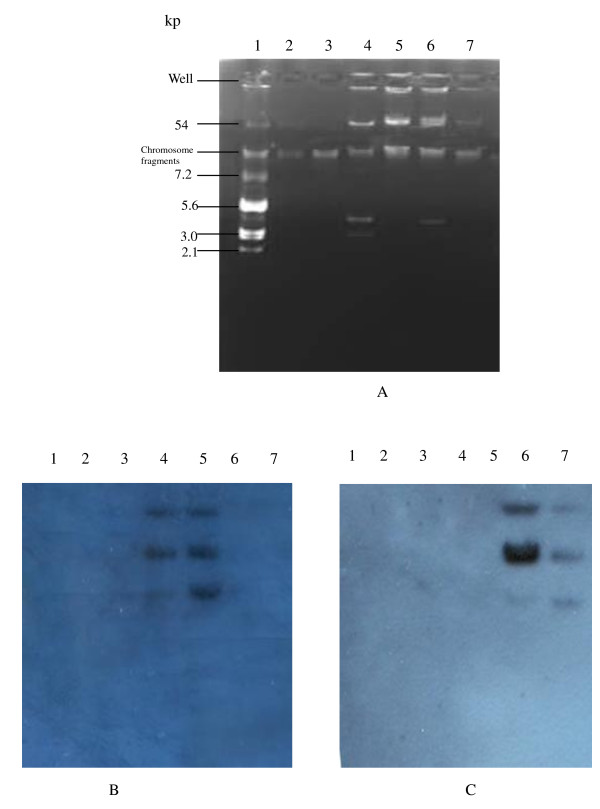
**Plasmid extractions from reference strains of *E. coli and clinical *isolates carrying *armA *or *rmtB *and their transformants (A), Southern hybridization with an *armA*-specific probe (B) and with a *rmtB*-specific probe (C)**. Lanes:1, *E. coli *V517; 2. *E. coli *V517/R1; 3, Amikacin-susceptible *E. coli *isolate; 4, E31; 5, E31 transformant; 6, E35; 7, E35 transformant.

High-level aminoglycoside resistance could be transferred by conjugation with frequencies between 10^-4 ^and 10^-6 ^only from four *rmtB*-positive donors through repeat attempts including using filter mating. The plasmids of the remaining 33 isolates with 16S rRNA methylase genes were transferred into the recipients, *E. coli *DH5α, by transformation. The transconjugants and transformants were highly resistant to amikacin, gentamicin and tobramycin (MICs, ≥256 μg/mL). The results of PCR and sequence analyses revealed that *bla*_TEM-1, _*bla*_CTX-M-14 _and *bla*_CTX-M-15 _were co-transferred with *rmtB *on plasmids to the recipients (Table 1). The plasmids of incompatibility groups IncF, IncK and IncN were detected in 34, 3 and 3 isolates, respectively, among which both IncF and IncK were detected in three isolates. The *armA*-positive (*E. coli *E31) and *rmtB*-positive (*E. coli *E35) isolates were selected for plasmid analyses. *E. coli *E31 contained three plasmids including 54-, 3- and 2-kb plasmids whereas *E. coli *E35 contained a 54-kb plasmid and a 3-kb plasmid (Fig. 1A). The 54-kb plasmids of *E. coli *E31 and its transformant hybridized with the *armA-*specific probe (Fig. 1B). The hybridization signals for a *rmtB*-specific probe were obtained on the 54-kb plasmids of the *E. coli *E35 harboring *rmtB *and its transformant (Fig. 1C). The DNA of *E. coli *V517, V517/R1 and amikacin-susceptible *E. coli *isolates had no hybridization signals with *rmtB*- and *armA*-specific probes.

### Genetic environments of the *armA *and *rmtB *gene

**Figure 2:**
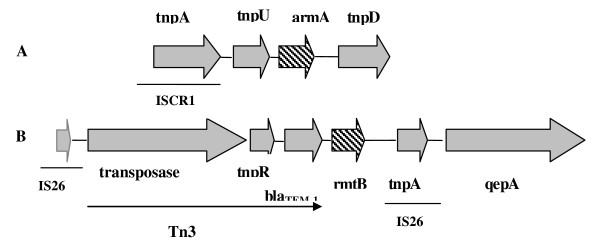
**Genetic environments of *armA *(A) and *rmtB *(B) genes**.

Upstream sequences of the *armA *gene contained *IS*CR1 and the putative transposase gene, *tnpU*. Another putative transposase gene, *tnpD*, was located downstream of the *armA *gene (Fig. 2A). The structural gene for the *rmtB *was preceded by a transposon, *Tn3*, including *tnpA *encoding transposase, *tnpR *encoding resolvase, and *bla*_TEM-1 _encoding spectrum beta-lactamase. A transposase gene, *tnpA*, and a quinolone efflux pump gene, *qepA*, were located downstream of the *rmtB *gene. The *rmtB *gene was flanked by two transposase genes comprising insertion sequence (IS26). Flanking regions of the *rmtB *gene are exhibited in Fig. 2B.

### PFGE

Thirty-seven 16S rRNA methylase gene-positive isolates distributing 19 different clinical units were grouped into 19 clonal patterns by PFGE, designated PFGE type A to S (Table 1). The predominant PFGE type was type H which accounted for 32.4% (12/37), followed by type A (3/37), type N (3/37), type G (2/37), type I (2/37) and type P (2/37). The remaining PFGE types were represented by a single isolate. The 12 PFGE type H isolates distributed nine different wards, whereas eight isolates isolated from the same ward (ward 11) belonged to seven separate PFGE types.

## Discussion

Although resistance rates of the *E. coli *isolates to gentamicin and tobramycin were more than 50.0% in our study, resistance to amikacin was relatively low. Since the *armA *gene was first identified in *K. pneumoniae *BM4536 isolated from a French patient in 2000 and the *rmtB *gene was initially found in *Serratia marcescens *S-95 isolated from a Japanese patient in 2002, these two genes have been found in Enterobacteriaceae, *Pseudomonas aeruginosa*, and *Acinetobacter baumannii *in many areas and particularly in Asia [4-6,14,17,22-24]. In the present study, the overall prevalence (5.4%) of 16S rRNA methylase genes in *E. coli *clinical isolates is higher than those previously reported in a Taiwanese study (0.4%) and a study from Shanghai, China (3.4%) [5,14]. Although *rmtA*, *rmtC*, and *npmA *have been detected in *P. aeruginosa*, *Proteus mirabilis*, and *E. coli*, respectively, in Japan [7,8,10], these genes were not detected in other Asian countries. Previous reports indicate a higher prevalence of *armA *relative to *rmtB *for Gram-negative bacilli [5,22,25,26]. One study reported that only *armA *was found in imipenem-resistant *A. baumannii *isolates from China [27]. However, *armA *was only detected in one *E. coli *isolate from our study, and our data are consistent with other studies indicating that *rmtB *may be more prevalent than *armA *among Enterobacteriaceae isolates and, in fact, that *rmtB *is the most prevalent 16S rRNA methylase gene among Enterobacteriaceae isolates in China [14,17]. This report further highlights the wide dissemination of 16S rRNA methylase genes among Enterobacteriaceae, and the spread of 16S rRNA methylase genes has become a great concern for hospitalized patients.

16S rRNA methylase genes have been linked to other resistance determinants, such as *bla*_TEM-1_, *bla*_CTX-M-3_, *bla*_CTX-M-14_, *sul1*, and *dfrXII*, and co-transferred with other resistance determinants on self-transferable plasmids to recipients by conjugation [5,28]. Similar to previous studies[4,5,22], most of 16S rRNA methylase gene-positive *E. coli *isolates reported in the present study were found to harbor *bla*_CTX-M _genes encoding ESBLs and were, therefore, resistant to multiple antibiotics. However, aminoglycoside resistance was not transferred by conjugation from 33 of 37 isolates harboring 16S rRNA methylase genes. Galimand *et al*. reported that the *armA *gene was part of functional composite transposon Tn*1548 *in plasmid pIP1204 [28]. The *rmtB *gene was found in the flanking region of the Tn*3*-like structure [6]. The genetic environment of the *armA *gene reported here was similar to that in *K. pneumoniae *reported in our previous study in which *IS*6100 and *IS*CR1 were first found to be located upstream of the *armA *gene [17], indicating that a similar mechanism may be responsible for the transfer of *armA *between *K. pneumoniae *and *E. coli *isolates in our hospital. The flanking regions of the *rmtB *gene in the present study were similar to that in previous reports in which *qepA *encoding quinolone efflux was located downstream of *rmtB *[29,30], but were different from those among in *K. pneumoniae *isolates in our previous report in which *traI *encoding conjugal nickase and helicase involved in conjugation were located downstream of *rmtB *[17]. The *armA *gene was usually carried by conjugative plasmids of replicon groups IncL/M and the *rmtB *gene was mainly associated with IncA/C and IncF conjugative plasmids [22,23,28]. In the present study, *rmtB *was borne by IncF plasmids in the majority of isolates. A small clonal dissemination was found, which accounted for 12 PFGE type H isolates. Based on our PFGE data, most 16S rRNA methylase gene-positive isolates exhibited only distantly related patterns and were from separate clinical units, suggesting considerable molecular heterogeneity amongst these isolates.

## Conclusion

High prevalence of plasmid-mediated *rmtB *gene was found among clinical *E. coli *isolates from a teaching hospital in China. The majority of 16S rRNA methylase gene-positive isolates also produced CTX-M type ESBLs. Both horizontal gene transfer and clonal spread were responsible for the dissemination of the *rmtB *gene.

## Competing interests

The authors declare that they have no competing interests.

## Authors' contributions

FYY, DY, CC, LHY and QQL performed the laboratory measurements. DQ and LXW made substantial contributions to conception and design. CP and DQ revised the manuscript critically for important intellectual content. ZQQ performed the analysis and interpretation of data. ZQQ, JYP and XQZ participated in design and coordination. FYY drafted the manuscript. All authors read and approved the final manuscript.

## Pre-publication history

The pre-publication history for this paper can be accessed here:

http://www.biomedcentral.com/1471-2334/10/184/prepub
